# Functional characterization of SMN evolution in mouse models of SMA

**DOI:** 10.1038/s41598-019-45822-8

**Published:** 2019-07-01

**Authors:** Erkan Y. Osman, Madeline R. Bolding, Eric Villalón, Kevin A. Kaifer, Zachary C. Lorson, Sarah Tisdale, Yue Hao, Gavin C. Conant, J. Chris Pires, Livio Pellizzoni, Christian L. Lorson

**Affiliations:** 1 Department of Veterinary Pathobiology, College of Veterinary Medicine, University of Missouri, Columbia, MO 65211 USA; 2 Bond Life Sciences Center, University of Missouri, Columbia, MO 65211 USA; 3 Center for Motor Neuron Biology and Disease, Department of Pathology and Cell Biology, Columbia University, New York, NY 10032 USA; 4 Bioinformatics Research Center, North Carolina State University, Raleigh, NC 27695 USA; 5 Division of Animal Sciences, University of Missouri, Columbia, MO 65211 USA; 6 Division of Biological Sciences, Christopher S. Bond Life Sciences Center, University of Missouri, Columbia, MO 65211 USA; 7 Department of Biological Sciences, Program in Genetics, North Carolina State University, Raleigh, NC 27695 USA

**Keywords:** Neurodegeneration, Molecular neuroscience

## Abstract

Spinal Muscular Atrophy (SMA) is a monogenic neurodegenerative disorder and the leading genetic cause of infantile mortality. While several functions have been ascribed to the SMN (survival motor neuron) protein, their specific contribution to the disease has yet to be fully elucidated. We hypothesized that some, but not all, *SMN* homologues would rescue the SMA phenotype in mouse models, thereby identifying disease-relevant domains. Using AAV9 to deliver Smn homologs to SMA mice, we identified a conservation threshold that marks the boundary at which homologs can rescue the SMA phenotype. Smn from *Danio rerio* and *Xenopus laevis* significantly prevent disease, whereas Smn from *Drosophila melanogaster*, *Caenorhabditis elegans*, and *Schizosaccharomyces pombe* was significantly less efficacious. This phenotypic rescue correlated with correction of RNA processing defects induced by SMN deficiency and neuromuscular junction pathology. Based upon the sequence conservation in the rescuing homologs, a minimal *SMN* construct was designed consisting of exons 2, 3, and 6, which showed a partial rescue of the SMA phenotype. While a significant extension in survival was observed, the absence of a complete rescue suggests that while the core conserved region is essential, additional sequences contribute to the overall ability of the SMN protein to rescue disease pathology.

## Introduction

Spinal Muscular Atrophy (SMA) is a debilitating neurodegenerative disease that manifests in a loss of α-motor neurons resulting in progressive muscle wasting, skeletal muscular atrophy and weakness. SMA is an autosomal recessive disease with a carrier frequency of 1:50 and an estimated incidence of 1/10,000 live births^1–4^. SMA is caused by deletion or mutation of the *survival motor neuron-1* gene (*SMN1*)^5^. All humans have a second, nearly duplicate gene to *SMN1*, known as the *survival motor neuron-2* (*SMN2*). The key difference between these genes is their propensity to promote differently spliced transcripts: *SMN1* produces 100% full length transcripts resulting in functional SMN protein, whereas *SMN2* is spliced such that 90% of transcripts lack exon 7, resulting in an unstable SMNΔ7 protein^6,7^. SMN is ubiquitously expressed and lack of SMN results in early embryonic lethality, while motor neurons are especially susceptible to a reduction in the levels of SMN that is characteristic of SMA^7^.

SMN plays key roles in RNA regulation through its diverse functions in the assembly of RNA-protein complexes^8^. SMN functions in the context of a multi-subunit macromolecular complex containing Gemin2–8 and Unrip to assemble heptameric rings of Sm and Lsm proteins on small nuclear RNAs of Sm class spliceosomal snRNPs that function in pre-mRNA splicing^9,10^ and U7 snRNP that participates in 3′-end processing of histone mRNAs^7^. SMN has also been implicated in the assembly of other RNP complexes through less characterized mechanisms^8,11^.

This study was designed to leverage the evolutionary distinct *SMN* homologs in order to identify conserved regions that rescued the SMA phenotype and provide insight into the SMA-linked functions of the SMN protein.

## Results

### Evolutionary conservation of SMN homologs

**Figure 1:**
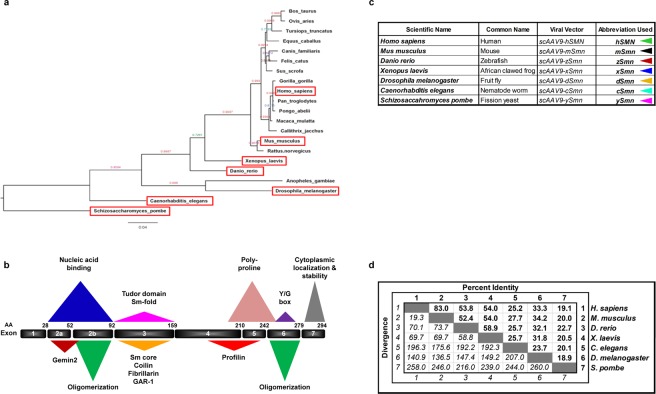
SMN conservation, functional domains, and homology across species. (**a**) The 50% majority-rule consensus tree was generated from Bayesian analysis of 21 protein sequences encoded by SMN orthologues (see Methods). The value at each branch is the Bayesian posterior probability for the split. *Schizosaccharomyces pombe* was assumed to be an out-group for rooting purposes. (**b**) The SMN protein map is divided into exon regions labeled with exon number and corresponding amino acid (AA) above. Domains of SMN that have been identified by their sequence characteristics and/or binding partners are labeled and defined by colored triangles. (**c**) Nomenclature of the species examined in the study. Scientific names, corresponding viral vectors and the appropriate abbreviations used. (**d**) Percent identity and evolutionary divergence of the species examined. Conservation relationships among tested SMN homologs. The percent identity is calculated from amino acid sequence differences. Divergence values represent information established from the phylogenetic relationship between the species.

SMN is a multifaceted protein with a number of interacting and functional domains that have been well-characterized, including self-oligomerization nucleic acid binding, binding to Gemins, and a scaffold for Sm core formation^8,12,13^. We identified relevant homologs of *SMN* that represent a spectrum of conservation levels. The chosen homologs were *Danio rerio, Xenopus laevis, Caenorhabditis elegans, Drosophila melanogaster*, and *Schizosaccharomyces pombe* (*zSmn*, *xSmn*, *cSmn*, *dSmn*, and *ySmn*, respectively) (Fig. 1). These organisms were selected because they represent a broad range of evolutionary distances and each SMN sequence has been previously characterized so that no hypothetical protein sequences were used^14–26^. These homologs maintain various degrees of identity at the amino acid level (Fig. 1). Moreover, phylogenetic analysis demonstrates that homologs from human and mouse share a node, those from zebrafish and frogs share a different node, and those from worms, flies, and yeast each have their own group (Fig. 1a). Analysis of the primary SMN sequences revealed a range of conservation from 83% to 18.9% identity (Fig. 1d). Furthermore, there are regions of high conservation, such as exon 2, 3, and 6 (Supplementary Fig. 1). These most highly conserved regions of SMN correspond to the N-terminal Gemin2 binding domain, the central Tudor domain, and the C-terminal YG box (Fig. 1b and Supplementary Fig. 1)^13,27,28^. Interestingly, y*Smn* is the only homolog that does not have a Tudor domain, which has been implicated in Sm protein binding and snRNP assembly^29,30^.

### *Effects of SMN* homologs on survival and weight gain in SMNΔ7 SMA mice

**Figure 2:**
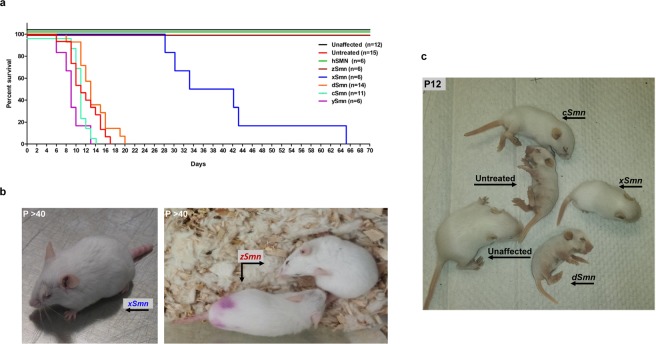
Delivery of SMN homologs results in variable lifespan extension in a severe mouse model of SMA. Viral vector injections for each tested SMN homolog were administered by ICV on P2 and life span was recorded. (**a**) Comparison of average survival time by Log-rank Mantel-Cox test. Kaplan-Meier survival curve depicts life span of healthy (unaffected), SMA (untreated) mice in comparison to SMN homologs-treated SMA mice. (**b**) Representative images of SMN∆7 mice (*Smn*^−/−^*;SMN2*^+/+^*;SMN∆7*^+/+^) injected with *xSmn* (left panel) and *zSmn* (right panel) homologs, respectively, past the age of P40. The treated mice display noticeable distal necrosis, which is exaggerated on the tail, eyelids and/or ears. (**c**) Representative image of SMN∆7 mice injected with *xSmn*, *cSmn*, *dSmn* homologs compared to control littermates at P12. The *xSmn* injected mouse has the appearance and gait of the unaffected littermate, while the *dSmn* and *cSmn* injected animals show slower growth and development delay similar to the untreated SMA mouse.

To test the relative efficacy of each homolog *in vivo*, *SMN* homologues were cloned into a vector backbone for production of self-complementary adeno-associated virus type 9 (scAAV9) delivery system. Previous studies showed that scAAV9-mediated human SMN expression in neonatal SMA mice can provide robust rescue of the SMNΔ7 mouse^31–34^. Therefore, a similar delivery paradigm was utilized to deliver the *SMN* homologs into the SMNΔ7 mouse model of SMA at postnatal day 2 (P2). Following a single injection of each vector, we monitored survival of SMA mice and found a variety of responses between the treatment groups (Fig. 2a). The previously described scAAV9-SMN expressing human *SMN* that was used as a positive control resulted in a significant extension in survival as expected^33–35^. Remarkably, delivery of *zSmn* resulted in a robust extension in survival, as all treated animals were alive at the completion of the study (P70) (Fig. 2a). Phenotypically, the *zSMN*-treated mice were active and mobile, and had well-maintained coats; however, distal necrosis was observed in the tail of some animals (Fig. 2b). A significant extension in survival was also observed in *xSmn*-treated animals, which displayed a median survival of 38 days and the longest-lived animal survived up to P65 (Fig. 2a). The longer-lived *xSmn*-treated animals showed distal necrosis of the tail and ears, their fur coats were unkempt, and kyphosis was present (Fig. 2b). These animals demonstrated tremors when walking and were less mobile than their healthy cohorts. The observed symptoms are all consistent with late stage phenotypes observed in intermediate mouse models of SMA or mice treated with other SMN targeting therapeutics^36,37^. Importantly, all the other *Smn* homologues were significantly less effective than either *xSmn* or *zSmn* (Fig. 2a,c). *ySmn* is the most divergent from human *SMN* and this cohort showed several early deaths; therefore, *ySmn* was assessed for overt toxicity in the healthy control animals. Unaffected animals were injected with the standard dosing of *ySmn* and monitored for several weeks, but none of the injected animals displayed an overt toxic response (data not shown).

**Figure 3:**
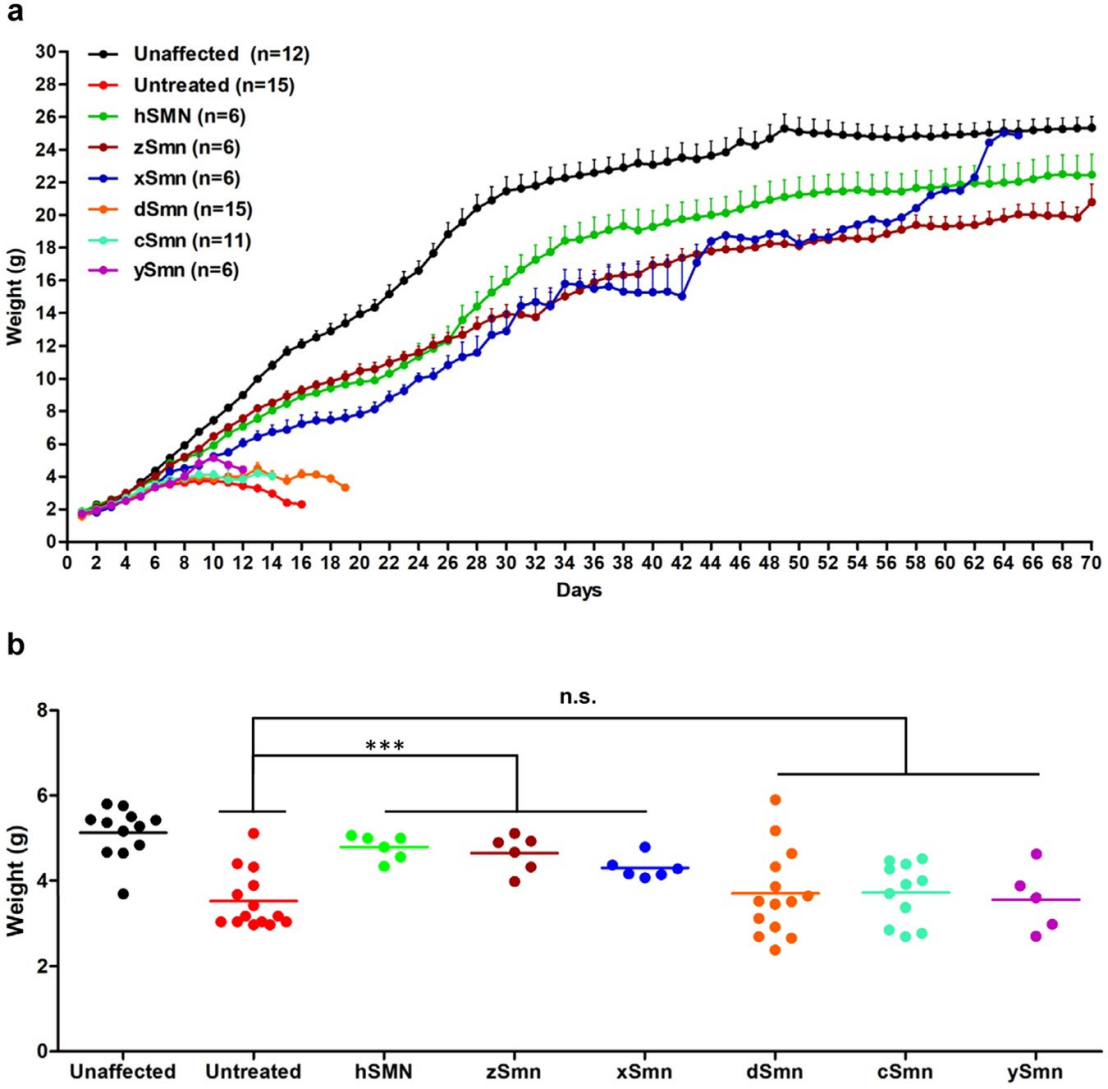
SMN homologs induce healthy weight gain in SMNΔ7 animals. (**a**) Weight gain curves in grams of SMNΔ7 mice treated with various SMN homologs and control littermates. Error bars indicate SEM. Statistical analyses were performed by Student’s *t*-test (of each treatment), where significance was shown with p < 0.0001 for untreated vs. hSMN, zSMN and xSMN cohorts; where no significant weight gain difference was observed between untreated and dSMN (p = 0.0681), untreated and cSMN (p = 0.2813), untreated and ySMN (p = 0.2874). (**b**) Individual body weights from all cohorts on P7. The scatter plot shows two divergent groups of SMN homologs that are distinct at earlier stages of life.

We next analyzed the effect of each treatment on weight gain. Animals treated with human *SMN* showed significant and continual weight gain (Fig. 3a). Consistent with the life extension data, *zSmn* and *xSmn* treated cohorts gained weight in a manner similar to human SMN treated SMA mice (Fig. 3a). In contrast, SMA mice treated with *dSmn*, *cSmn*, and *ySmn*, which were the three groups that failed to significantly rescue life span (Fig. 2a), did not significantly differ from untreated SMA mice in weight gain (Fig. 3a,b). Collectively, these results demonstrate that *zSmn* and *xSmn* provide significant protection from disease development, delineating those species that are more distantly related and less capable of preventing the SMA phenotype.

### *Expression of zSmn* and *xSmn* increases motor function in SMA mice

**Figure 4:**
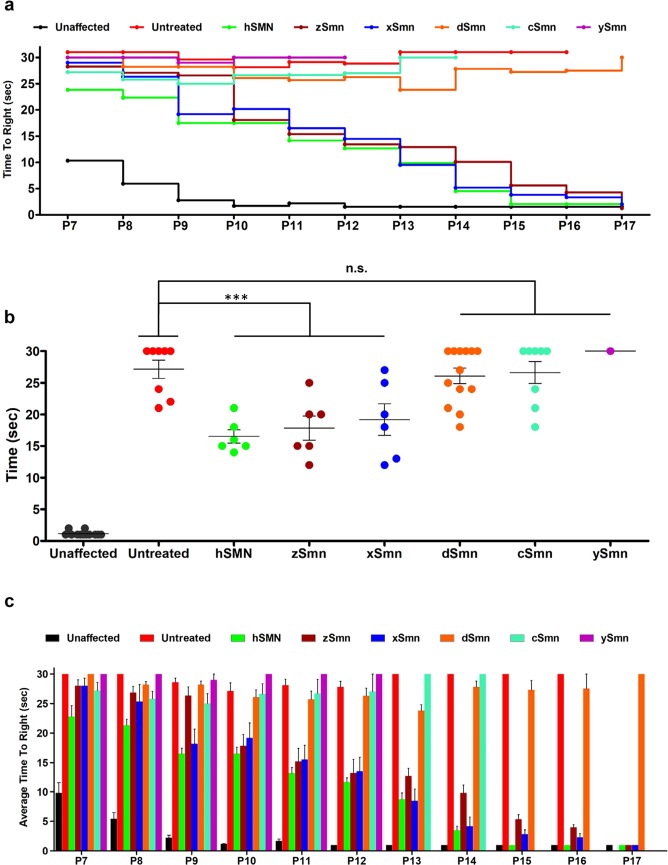
Assessment of motor function of treated SMNΔ7 mice after treatment with various SMN homologs. (**a**) Line graph representing raw data of the average time to right from P7 to P17. Animals injected with *hSMN*, *zSmn* and *xSmn* displayed significant improvement in motor function through their life span. (**b**) Scatter plot of time-to-right ability of treated cohorts highlights the difference in performance for each individual mouse (values are shown for P12). Statistical analysis was carried out by one-way ANOVA and significance is represented by “*”p ≤ 0.05; “**”p ≤ 0.01; “***”p ≤ 0.001; and “n.s.”p > 0.05. (**c**) Bar graph showing the average time-to-right in seconds of each treatment group from ages of P7 through P17, where error bars represent significance compared to the control groups. Data expressed as S.E.M.

To determine the effects of AAV9-mediated expression of SMN homologues on motor function, we performed the time to right assay (TTR)^38^ starting at one week of age (P7). While healthy animals can readily right themselves, SMNΔ7 SMA mice are rarely able to complete this task and the attempt is recorded as “failed” after 30 seconds (Fig. 4a). Consistent with survival and weight gain patterns, *zSmn* and *xSmn* treatment groups performed similar to human SMN treated SMA mice and significantly faster than *dSmn*, *cSmn*, and *ySmn* cohorts (Fig. 4a–c). By P17, all *zSmn-* and *xSmn-*injected animals were able to right themselves. In contrast, only a few animals injected with *cSmn* were able to right themselves. Animals from *dSmn* and *ySmn* cohorts displayed little motor function and did not successfully right (Fig. 4c and Supplementary Fig. 2). Collectively, these results demonstrate that *zSmn* and *xSmn* homologs are able to significantly rescue the SMA phenotype in SMNΔ7 mice, while expression of *cSmn*, *dSmn*, and *ySmn* are unable to rescue important hallmarks of disease, ranging from life span to motor function. These results suggest that a significant conservation threshold exists between *X. laevis* and *C.elegans* homologs, indicating that SMN has acquired relevant domains for its function in vertebrates that are missing in lower species in the evolutionary scale.

### Correction of neuromuscular junction pathology by divergent SMN homologs

**Figure 5:**
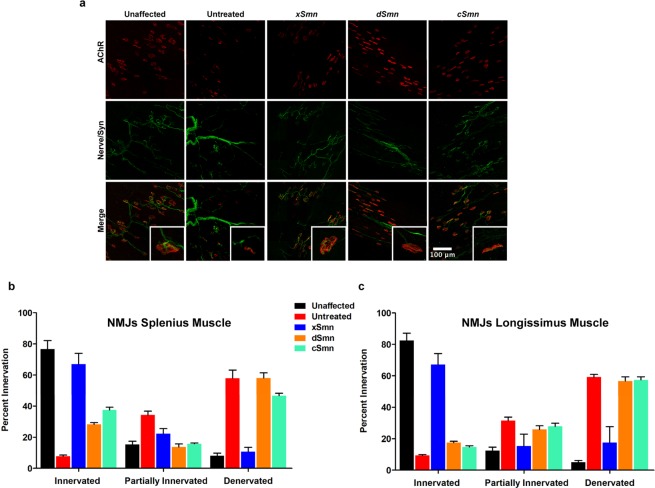
Neuromuscular junction pathology in SMN∆7 mice after delivery of SMN homologs. (**a**) Representative immunohistochemistry of NMJs of individual cohorts injected with xSmn, dSmn, cSmn and compared to the age-matched controls. Neurofilament and synaptic vesicle are shown in green. Acetylcholine receptors are stained with α-Bungarotoxin (red). NMJs were analyzed from the longissimus capitis (shown) and the splenius muscles, harvested from cohorts at P12. (**b**) Quantification of percent NMJ innervation in the splenius muscle. (**c**) Quantification of percent NMJ innervation in the longissimus muscle. Data was analyzed using by one-way ANOVA and statistical significance is represented by “*”p ≤ 0.05; “**”p ≤ 0.01; “***”p ≤ 0.001; “****”p ≤ 0.001; “n.s.”p > 0.05 and expressed as S.E.M.

Based on the data of phenotypic suppression described above, we further analyzed three constructs that were closely related and spanned the conservation threshold: *xSmn*, *dSmn*, and *cSmn*. Specifically, we investigated the capacity of these SMN homologs to correct neuromuscular junction (NMJ) structure and integrity, which represent a clinically-relevant SMA phenotype. During normal NMJ development, motor endplates undergo specific stages of maturation that are characterized by plaque, perforated, C-shaped, branched, and pretzel-like shapes at the morphological level^39,40^. NMJs are disrupted in multiple mouse models of SMA, including the SMNΔ7 model, and NMJ pathology in SMA can take the form of pre-synaptic neurofilament accumulation, post-synaptic immaturity, or denervation of motor endplates depending on the specific degree of vulnerability of distinct muscles to SMN deficiency^39–41^. Therefore, we looked for morphological abnormalities in the NMJs of two vulnerable muscle groups at P12: splenius and longissimus. NMJs from SMA mice treated with *cSmn* and *dSmn* showed significant defects in the motor endplate (Fig. 5a). Most of the motor endplates appeared developmentally delayed, displaying plaque and perforated morphologies, and showed lower levels of innervation compared to controls (Fig. 5b,c). In contrast, animals treated with *xSmn* had an NMJ profile that was qualitatively similar to the unaffected cohort (Fig. 5a), resulting in similar levels of innervation and denervation (Fig. 5b,c). These results indicate that the *xSmn* homolog prevents NMJ developmental defects with significantly higher efficacy compared to *dSmn* or *cSmn*.

### Functional analysis of SMN homologs in RNA processing

**Figure 6:**
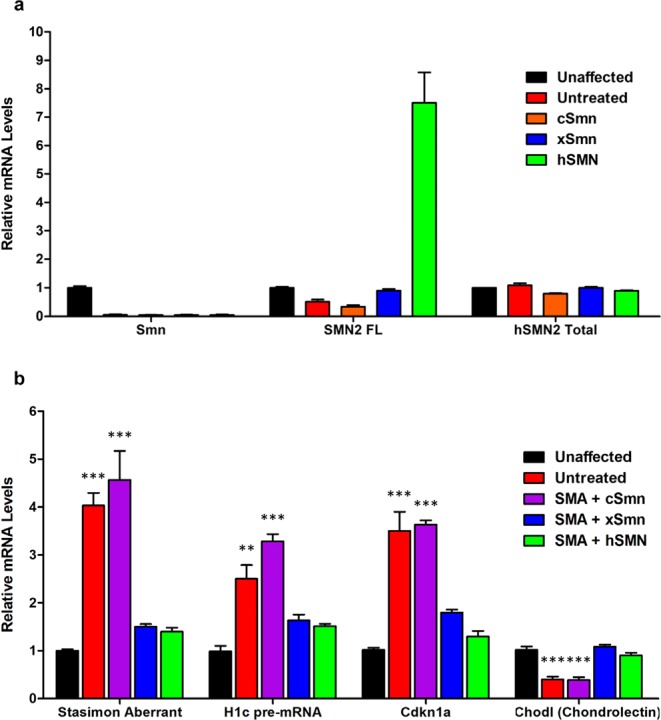
Functional analysis of SMN homologs in RNA processing. Total mRNA levels from mouse spinal cord tissues (n = 3) were assayed by RT-qPCR. Relative mRNA levels are normalized and compared to unaffected wild type animals. (**a**) Levels of total endogenous mouse *Smn*, full-length (SMN2 FL) and total human SMN transcripts from the SMN2 gene. (**b**) Analysis of representative SMN-dependent RNA processing events that are dysregulated in SMA mice, which include aberrant U12 splicing of Stasimon mRNA, 3′-end processing of H1c histone mRNA, accumulation of Cdkn1a mRNA, and decreased Chodl mRNA expression. *cSmn* is not able to correct mRNA processing defects induced by SMN deficiency in SMA mice (untreated, red), whereas *xSmn* rescue of these defects was as effective as treatment with full-length human SMN (hSMN). Data represent mean and S.E.M from independent biological replicates. Statistical analysis was carried out by one-way ANOVA and significance is represented by “*”p ≤ 0.05; “**”p ≤ 0.01; “***”p ≤ 0.001.

To determine the functionality of SMN homologs at the molecular level, we analyzed their ability to correct specific RNA processing defects in diverse pathways that are regulated by SMN and disrupted in SMA^8^. First, we monitored by RT-qPCR the mRNA levels of endogenous mouse *Smn* as well as full-length and total human SMN2 transcripts in the spinal cord of control and AAV9-treated SMA mice as previously described^42^. The AAV9 vectors did neither change the level of the endogenous *Smn* gene nor total *SMN* expression from the *SMN2* transgene (Fig. 6a), while exon 7-containing full-length SMN transcripts were only increased in samples derived from spinal cord of SMA mice with scAAV9 expressing human SMN cDNA-derived transcripts as expected (Fig. 6a). Next, we analyzed the ability of each SMN homologue to correct RNA processing defects induced by SMN deficiency using a previously established panel of representative mRNAs that serve as markers of distinct SMN-dependent RNA pathways^8^. Specifically, we monitored aberrant U12 splicing of the *Stasimon* gene as a readout of minor splicing dysfunction^43^ as well as defective 3′-end processing of histone H1c mRNA caused by impairment of SMN-mediated assembly of U7 snRNP^7^. We also analyzed accumulation of Cyclin-dependent kinase inhibitor 1 A (Cdkn1a) mRNA resulting from p53 activation induced by dysregulation of Mdm2 and Mdm4 alternative splicing in SMA mice^44,45^. Lastly, we monitored expression of Chondrolectin (Chodl) mRNA, a motor neuron-specific gene that is expressed at lower levels in SMA mice^46–49^ due to altered miRNA regulation^50^. To perform this analysis, animals that received each treatment were harvested at P12 and their spinal cords were used to assess mRNA changes (Fig. 6b). Consistent with previous studies, SMA mice showed accumulation of aberrantly spliced Stasimon mRNA, 3′-end extended histone H1c mRNA, and Cdkn1a mRNA as well as decreased levels of Chodl mRNA relative to unaffected controls (Fig. 6b). Tcs Importantly, *xSmn* was able to correct all of these RNA processing events to an extent similar to human SMN (Fig. 6b). In contrast, *cSmn* did not yield any correction of mRNA changes compared to SMNΔ7 mice (Fig. 6b). These results demonstrate that the correction of RNA processing brought about by SMN homologs correlates with their ability to suppress the SMA phenotype in this mouse model.

### Identification of a minimal functional domain in SMN

**Figure 7:**
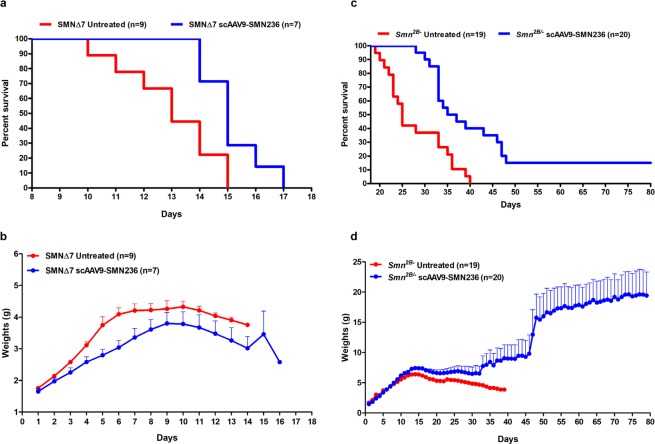
AAV delivery of SMN236 – an artificial protein containing only the most highly evolutionarily conserved domains of human SMN – improves survival and weight gain in SMA mice. SMN homologs demonstrate regions of high conservation in the amino acid sequences encoded by human SMN exons 2a, 2b, 3 and 6. Therefore, the SMN236 construct was designed to contain these regions and incorporated into a viral vector for gene delivery in mouse models of SMA. (**a**,**b**) Analysis of SMN236 injected in the severe SMNΔ7 mice on survival (**a**) and weight gain (**b**) compared to the age-matched control littermates. The difference in survival between treated (median 15 days) and untreated SMNΔ7 (median 13 days) mice was calculated by the log-rank Mantel-Cox test (p = 0.015). (**c**,**d**) SMN236 delivered into the intermediate *Smn*^*2B/−*^ mouse model of SMA delayed early deaths with median survival 25 days for untreated vs. 36 days for the treated animals (Mantel-Cox test; p = 0.00023) (**c**) and has the greatest weight gain effect in the long-lived animals (**d**). Data expressed as mean and S.E.M.

To determine the regions of SMN that could mediate the rescuing activity observed in the previous experiments, we utilized a multiple sequence alignment to identify the highest conserved regions between the rescuing homologues (Supplementary Fig. 1). To test whether conserved domains are sufficient to act as a minimal functional domain for *SMN* while maintaining the human *SMN* amino acid sequence, we created a synthetic, human SMN-based sequence that comprised amino acids encoded by exons 2a, 2b, 3, and 6 (named SMN236). Utilizing the same scAAV delivery strategy, we delivered scAAV9-eGFP:SMN236 into SMNΔ7 SMA animals that expresses a GFP protein fused to the amino-terminus of SMN236. Delivery of this vector to the severe SMNΔ7 model did not increase weight gain, but prevented the earlier deaths observed in untreated SMA mice such that the first death occurred at P14 compared to P10 (Fig. 7a,b). While robust therapeutics are able to rescue this model^34,51–54^, some compounds cannot overcome the severe and rapid decline seen in SMNΔ7 animals. Therefore, we also examined the minimal domain construct in a less severe model of SMA, the *Smn*^*2B/−*^ mouse^36^. Similar to the results in the severe mouse model, the earliest deaths were shifted from P19 to P28 (Fig. 7c). Furthermore, the overall life span was significantly extended with an increase of the median survival from 25 to 36 days after treatment with SMN236 (Fig. 7c). Consistent with the life span extension, SMN236 treatment resulted in significant weight gain compared to the untreated cohorts, all of which failed to reach 5 grams of total body weight, while several treated *Smn*^*2B/−*^ mice achieved 10–15 grams of total body weight (Fig. 7d). While these improvements in the SMA phenotype were statistically significant, it is worth noting that treatment with SMN236 was not a complete rescue, suggesting that additional regions within the SMN protein are required for full restoration of SMA-associated functions.

## Discussion

This work was designed to take a unique evolutionary approach to investigate the SMA-associated SMN function. Comparison of the human SMN amino acid sequences with the sequences of several other species allowed the visualization of highly conserved regions, leading to the inference of a minimal, but crucial functional domain of the SMN protein consistent with previous reports that highlight the functional significance of the 2,3,6 region^20,28,55–59^. Based on sequence conservation, it is reasonable to hypothesize that SMN exons 2, 3, and 6 are extremely important for SMN function and might be sufficient for SMN rescue. This is what was observed using a synthetic construct consisting exclusively of SMN exons 2,3, and 6, resulting in an extension in survival in two important models of SMA. However, it is also clear that this was not a complete rescue of the SMA phenotype. Biochemically, these regions are responsible for mediating a number of SMN properties, including self-oligomerization and interaction with core components of the SMN complex and RNP targets^13,27,60–63^, suggesting that this synthetic construct likely contains critical functions of the SMN protein and that partial functionality has been retained. These regions are also relative hot spots for SMA causing missense mutations, further highlighting their importance with respect to disease development.

This work leverages a novel approach to establishing a minimal functional domain based upon the analysis of evolutionarily conserved SMN regions. Further analysis could identify patterns aside from the simple conservation threshold required for a phenotypic improvement that we empirically determined to be between *xSmn* and *dSmn* based upon life span extension in SMA mice. First, there is a significant difference between the efficacy of *dSmn* and *xSmn* that we did not further investigate. It would be of interest to compare another non-mammalian vertebrate species to see whether the rescue effect afforded also falls within this gradient. Unfortunately, no reptilian SMN sequence has been characterized and therefore this work would rely on a predicted protein sequence. The sequential differences responsible for the differential rescue effect are not immediately apparent from sequence comparison. These differences likely do not represent a lack of function and would require more sensitive analysis to tease apart.

Another interesting suggestion within our data is the discrepancy between *xSmn* and *hSMN* with respect to survival. The SMN homologs from human, mouse, share only 54% and 58% identity with *Xenopus*, respectively, thus the differences in their effect *in vivo* are not overly surprising, although several SMN mutations act similarly in zebrafish models^64^. The rescue effect of *xSmn* was significant, but not nearly as effective as rescuing survival with human *SMN*. However according to the mRNA analysis, the *Xenopus laevis* homolog was able to effectively rescue SMN-dependent mRNA processing in SMA mice *in vivo*, implying that its function in RNA regulation was intact. This is consistent with previous findings that *xSmn* is capable of restoring Cajal body formation in an SMN knockdown environment^65^. Cajal body formation can only be restored by the proper import of nascent snRNP complexes^66^. It can then be inferred that the difference in the efficacy with *xSmn* is not due to an overt loss-of-RNP function, but perhaps a loss of function for specific mRNAs, or in combination with another SMA-associated function.

The minimal SMN construct, SMN236, which partially rescued the SMA phenotype, represents the most highly conserved regions of SMN that are also thought to be most critical for its function. While this construct was unable to fully rescue the severe or intermediate mouse models of SMA, it did result in a significant extension in survival. With such a highly mutated protein, we did not envision that all of the full-length SMN protein activity would be retained, yet this partial activity could be due to something as basic as protein folding. Alternatively, these results could imply that functions encoded within exons 1, 4, 5, or 7 may contain additional key elements for SMN function in SMA. Previously, mild missense SMN missense mutations have been shown to only rescue the SMA phenotype in the presence of full-length SMN produced by SMN2^67^, therefore, we may be observing a requirement for a 2,3,6/FL complex, but the levels of full-length are not sufficient to drive the formation of this heteromeric complex. While RNP assembly is an essential housekeeping function, it and other functions may combine to contribute to the complex motor neuron specific display of SMA symptoms. Overall, these results support the previous published work, that disruption of RNA processing plays a role in the pathogenesis of SMA^68^ and the deficiencies in SMN oligomerization correlate with the severity of the disease^69^.

## Methods

### SMN orthology and evolutionary tree

Mammalian orthologues sequences to human SMN gene were inferred using a previously described method^70^. SMN orthology for more distant species was verified using Ensembl^71^, except for SMN homologs from frog (*Xenopus laevis*), mosquito (*Anopheles gambiae*) and yeast (*Schizosaccharomyces pombe*), which were identified through a manual BLAST search (Supplementary Table 1). Orthologous SMN protein sequences were downloaded from UniProt^72^. Multiple sequence alignments and the phylogenetic trees were estimated using the Bayesian analysis software BAli-Phy version 3.4^73^, with substitution model lg08^74^ and indel model rs07^75^. Five MCMC chains of 50,000 iterations were run, and the first 10% samples of each chain were removed as burn-in. The tree samples were then combined to find a majority-rule consensus tree with the posterior probability of each split larger than 0.5. The resulting unrooted consensus tree was rerooted using FigTree v1.4.4^76^ and visualized using the R package ape 5.2^77^ in R version 3.4.1^78^.

### Animal procedures and experiments

All animal experiments were carried out in accordance with protocols approved by the University of Missouri Animal Care and Use Committee as well as the regulations established by the National Institute of Health’s Guide for the Care and Use of Laboratory Animals^79^. For the severe SMNΔ7 animal model, heterozygous breeder pairs of mice (*Smn*^*+/−*^*;SMN2*^+/+^*;SmnΔ7*^+/+^), were purchased from the JAX^®^ Laboratory (JAX®Stock#005025:FVB.CgGrm7Tg(SMN2)89AhmbSmn1tm1MsdTg(SMN2*delta7)4299Ahmb/J; The Jackson Laboratory, 610 Main Street Bar Harbor, ME 04609 USA). The *Smn*^*2B/–*^ mice were bred from two colonies: *mSmn*^*+/−*^ heterozygotes (stock no. 006214; Jackson Laboratory) and *Smn*^*2B/2B*^ homozygotes (C57BL/6 background; gracious gift from Dr. Rashmi Kothary, Ottawa Hospital Research Institute, Ottawa, Ontario, Canada). Animals were fed low-fat stock diets (Harlan Teklad 8640). The colony was maintained as heterozygote breeding pairs under specific pathogen free conditions. Experimental mice litters (*Smn*^−/−^*; SMN2*^+/+^*; SMNΔ7*^+/+^ referred as SMNΔ7^80^ and *Smn*^*2B/*^) were genotyped on the day of birth (P0) using standard PCR protocol (JAX® Mice Resources) on tail tissue material as previously described. Two sets of primer sequences (Supplementary Table 2) were used for the mouse *Smn* gene. Experimental knockout pups were kept with a minimum of two healthy heterozygous siblings. Wild type and additional heterozygous animals were culled to maintain control for litter size of 4–5 pups. All littermates from the experiments were weaned from their mother at age of 21 days and housed in cages with their siblings according to gender. Intracerebroventricular (ICV) injections were performed at P2 as previously described^81,82^. For motor function analysis, time to right (TTR) experiments were conducted as previously described^38,83^. TTR was measured every day from P7 through P17 on control and SMNΔ7 experimental animal cohorts. Mice were placed on their back and were given a maximum of 30 seconds to successfully turn themselves. Mice that did not right within 30 seconds were considered to have failed the test. At each occasion, three attempts were recorded separately and averaged.

### Viral preparation

Viral constructs were prepared as previously described^84,85^.Purified virus preps were dialyzed against a HEPES buffer (20 mM HEPES, 100 mM NaCl; Fisher Scientific Co. LLC; Hanover Park, IL 60133) and quantified using quantitative PCR. The final fractions were stored at 4 °C until use. All transgenes are driven under the control of the chicken beta actin (CBA) promoter unless otherwise stated.

### Real-time PCR

Quantification of viral genomes was performed using SYBR® Green Master Mix (Cat. 4385612; Applied Biosystems, Foster City, CA 94404) and primers to amplify the chicken β-actin promoter region forward (5′-CCGGTGGTGGTGCAAATCAAAGAA-3′) and reverse (5′-AGCAGAAGTAACACTTCCGTACAGGC-3′). The absolute quantitation method using a standard curve was utilized on the Applied Biosystems® 7500 Real-Time PCR system using the included Sequence Detection Software Version 1.3 (Applied Biosystems, Foster City, CA). Viral fractions were diluted 1:1000 and the PCR cycle was as follows: 50 °C 2 min, 95 °C for 10 min, 40 cycles (95 °C for 15 sec), 60 °C for 1 min. A standard curve was obtained using serial dilutions of the transgene-containing plasmid to calculate the melting curves of each sample. The viral fractions containing the highest titer were dialyzed with HEPES buffer. Following dialysis, qPCR was performed to obtain the final titer of the virus-containing solution to be used for injections into the experimental mice.

### Administration of viral vectors

All experimental mice were injected on P2 with 1 × 10^11^ viral genome particles (vgp) as determined by qPCR, unless otherwise specified. Each aliquot to be injected was mixed 1:50 with filtered green food dye (McCormick & Co., Sparks, MD 21152, USA) immediately prior to injection. Upon ICV injection, dye localization was used to determine the success of the injection and subjects that received sub-par injection were not used as data points. ICV delivery was performed using a pulled capillary sterile glass needles as previously described^82,85^. Each injection consisted of up to 6 μl total volume. Due to some variability in viral titer, some constructs were injected up to 3 times to achieve 1 × 10^11^ viral particles. In such instances, injections were spaced such that a minimum of 4 hours were allowed for uptake of the previous injection before administration of the next.

### RNA analysis

The total RNA was isolated from spinal cord tissue using Trizol (Thermo Fisher Scientific, Cat. # 15596-026) followed by digestion with RNase-free DNase I (Thermo Fisher Scientific, Cat. # AM2222). For mRNA analysis, mixture of oligo-dT primers and random hexamers was used to generate cDNA using Advantage^®^ RT-for-PCR kit (Clontech) and 1 μg of total RNA following the manufacturer’s instructions. All primers used in RT-qPCR experiments are listed in Supplementary Table 3 and were previously described^42–44^. SMN2 full-length (SMN2 FL) measures exon 7-included human SMN mRNA isoforms, including the mRNA expressed from the AAV9-hSMN cDNA. SMN Total represents human SMN2 total mRNA (irrespective of exon 7 splicing) since the primers are specific for the 3′ UTR of the human SMN2 transgene present in the SMNΔ7 mice^42^.

### Neuromuscular junction analysis

All NMJ procedures were performed as previously described^39,84^. Fluorescent images were taken on a Leica (Buffalo Grove, IL), using Leica Application Suite X (LAS^®^X) software. Confocal imaging was performed using a Zeiss LSM 510 META (Carl Zeiss Inc.; Thornwood, NY) confocal microscope. Z-stacked images were taken at 1μm intervals and deconvoluted using MetaMorph^®^ Imaging System software. Images presented are maximum projections of Z-stacked images. Muscle analysis was done by blinded counts for a minimum of 4 fields of view per muscle type from n = 3 animals per treatment.

### Statistical analysis

Statistical analysis and calculations were performed utilizing several software and analytical methods. Kaplan-Meier survival data for the study groups were analyzed with a Log-rank Mantel-Cox test (Graph-Pad Prism v5.00; GraphPad Software, Inc., 7825 Fay Avenue, Suite 230, La Jolla, CA 92037 USA). A p-value of ≤ 0.05 was considered statistically significant. For weight gain and TTR measurements statistical analysis were performed by GraphPad Prism as above (2-way ANOVA with Tukey’s multiple comparisons), by Student’s *t*-test using Microsoft Excel 2013, version 15.0.4753.1003 and by IBM SPSS Statistics software. Statistical analysis of RNA levels was carried out by one-way ANOVA followed by Tukeys’ post-hoc test using Graph-Pad Prism v5.00.

## Supplementary information

Supplementary Material
