# Supplementary material for: Functional characterization of SMN evolution in mouse models of SMA
Source: Sci Rep. 2019 Jul 1;9:9472. doi: 10.1038/s41598-019-45822-8 (PMC6603021; doi:10.1038/s41598-019-45822-8)
Supplement: Supplementary file 1 — Supplementary Material [file 41598_2019_45822_MOESM1_ESM.pdf]

Functional characterization of SMN evolution in mouse models of SMA

Erkan Y. Osman[1,2 ‡], Madeline R. Bolding [1,2 ‡], Eric Villalón [1,2], Kevin A. Kaifer [1,2], Zachary C. Lorson [1,2], Sarah Tisdale [3], Yue Hao [4], Gavin C Conant [4, 5, 6], J. Chris Pires [7], Livio Pellizzoni [3], and Christian L. Lorson [1, 2 *]

[1] Department of Veterinary Pathobiology, College of Veterinary Medicine, University of Missouri, Columbia, MO, 65211, USA

[2] Bond Life Sciences Center, University of Missouri, Columbia, MO, 65211

[3] Center for Motor Neuron Biology and Disease, Department of Pathology and Cell Biology, Columbia University, New York, NY 10032,

[4] Bioinformatics Research Center, North Carolina State University, Raleigh, NC, 27695

[5] Division of Animal Sciences, University of Missouri, Columbia, MO 65211,

[6] Division of Biological Sciences, Christopher S. Bond Life Sciences Center, University of Missouri, Columbia, MO, 65211

[7] Department of Biological Sciences, Program in Genetics, North Carolina State University, Raleigh, NC, 27695

Supplementary Figure 1

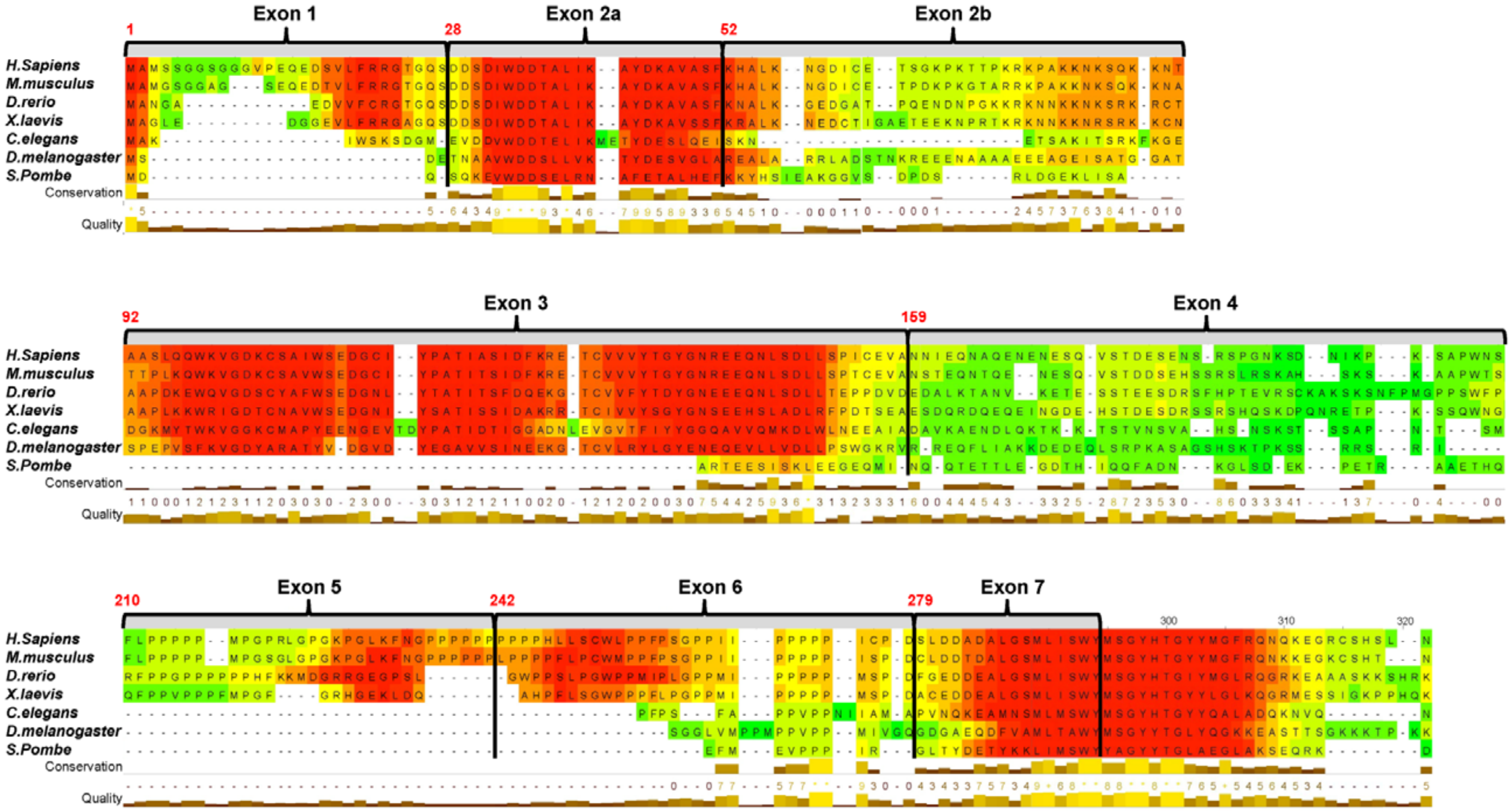

Supplementary Figure 1 Multiple alignment of SMN homologs demonstrates variable similarities and areas of conservation

Amino acid sequences from SMN homologs were aligned and visualized using a ClustalΩ (Omega) [43,44] all against all pair-wise alignments combined with LALIGN all against all local alignments via the T-Coffee® webserver [45-47]. Red areas demonstrate regions of high alignment confidence, whereas green indicates low confidence. Conservation levels are indicated by small bars under the alignments. Quality of alignments is demonstrated in the bottom row and indicated with a single digit number (0 through 10).

Supplementary Figure 2

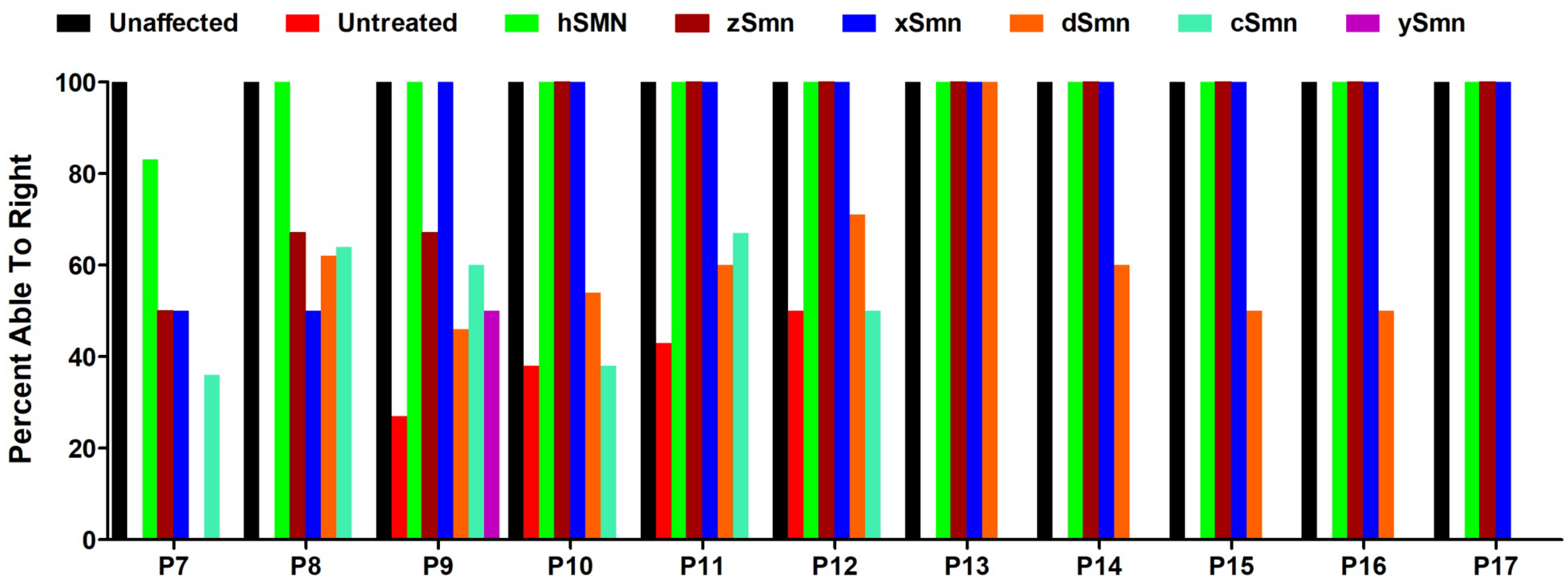

Supplementary Figure 2 Muscle functionality of SMNΔ7 mice injected with various SMN homologs
Bar graph showing the percent animals able to right themselves compared to the control littermates. Results shows that SMNΔ7 mice treated with full length *hSMN*, *zSmn*, and *xSmn* perform substantially better. Animals injected with *dSmn*, *cSmn*, and *ySmn* exhibit less muscle control and turn slower.

Supplementary Table 1

	Species	Common name	SMN Gene ID	Trascript ID	bp	Protein UniProt	Length	Ortholog verified	Source
1	*Felis catus*	Cat	ENSFCAG00000028292	ENSFCAT00000031796.3	885	R4GF28	294aa	Y	ORIS
2	*Canis familiaris*	Dog	ENSCAFG00000007812	ENSCAFT00000012477.3	2271	K9J6P1	289aa	Y	ORIS
3	*Equus caballus*	Horse	ENSECAG00000015215	ENSECAT00000016268.2	905	F6TME2	291aa	Y	ORIS
4	*Tursiops truncatus*	Dolphin	ENSTTRG00000005080	ENSTTRT00000005065.1	876	A0A2U4AWX6	291aa	Y	ORIS
5	*Bos taurus*	Cow	ENSBTAG00000005743	ENSBTAT00000007547.6	973	O18870	287aa	Y	ORIS
6	*Ovis aries*	Sheep	ENSOARG00000005425	ENSOART00000005910.1	822	W5P5W7	273aa	Y	ORIS
7	*Rattus norvegicus*	Rat	ENSRNOG00000018067	ENSRNOT00000024456.5	1226	Q6P684	288aa	Y	ORIS
8	***Mus musculus***	**Mouse**	**ENSMUSG00000021645**	**ENSMUST00000022147.14**	**1222**	**P97801**	**288aa**	**Y**	ORIS
9	*Pongo abelii*	Orangutan	ENSPPYG00000015533	ENSPPYT00000018065.2	1625	A0A0A0MXR7	276aa	Y	ORIS
10	***Homo sapiens***	**Human**	**ENSG00000172062**	**ENST00000380707.8**	**1536**	**Q16637**	**294aa**	**Y**	ORIS
11	*Pan troglodytes*	Chimpanzee	ENSPTRG00000016955	ENSPTRT00000084001.1	1067	A0A2I3TMK9	297aa	Y	ORIS
12	*Macaca mulatta*	Macaque	ENSMMUG00000020214	ENSMMUT00000028438.3	885	F6V985	294aa	Y	ORIS
13	***Xenopus laevis***	**Frog**	**AY048668.1**			**AAL07278.1**	**282 aa**	**-**	GenBank
14	***Danio rerio***	**Zebrafish**	**ENSDARG00000018494**	**ENSDART00000028099.7**	**1019**	**Q9W6S8**	**281aa**	**Y**	Ensembl
15	*Anopheles gambiae*	Mosquito	AGAP004011	AGAP004011-RA	1102	Q7Q266	313aa	-	VectorBase
16	***Drosophila melanogaster***	**Fruitfly**	**FBgn0036641**	**FBtr0329921**	**1086**	**Q9VV74**	**226aa**	**Y**	Ensembl
17	***Caenorhabditis elegans***	**Nematode**	**WBGene00004887**	**C41G7.1b**	**864**	**G5EFQ8**	**207aa**	**Y**	Ensembl, WormBase
18	***Schizosaccharomyces pombe***	**Yeast**	**NM_001018486.2**			**NP_593088.1**	**152 aa**	**-**	NCBI
19	*Sus scrofa*	Pig	ENSSSCG00000029127	ENSSSCT00000030798.2	753	I3LSV5	250aa	Y	ORIS
20	*Callithrix jacchus*	Marmoset	ENSCJAG00000009045	ENSCJAT00000017512.1		ENSCJAP00000016562	276aa	Y	ORIS
21	*Gorilla gorilla*	Gorilla	ENSGGOG00000026704	ENSGGOT00000029602.2	678	G3SHH7	225aa	Y	ORIS

Supplementary Table 1 SMN orthologs Ensembl gene IDs and corresponding UniProt protein IDs.

Supplementary Table 2

Genotyping Primers	Primer Sequences
mSmn -WT Forward	**5'-TCTGTGTTCGTGCGTGGTGACTTT-3'**
mSmn -WT Reverse	**5'-CCCACCACCTAAGAAAGCCTCAAT-3'**
SMN1-KO Forward	**5'-CCAACTTAATCGCCTTGCAGCACA-3'**
SMN1-KO Reverse	**5'-AAGCGAGTGGCAACATGGAAATCG-3'**

Supplementary Table 2 List of primer sets used for animal genotyping

Supplementary Table 3

Name	Forward Sequence (5' to 3')	Reverse Sequence (5' to 3')
**Cdkn1a**	GACATTCAGAGCCACAGGCACC	GAGCGCATCGCAATCACGGCGC
**Chodl**	CCTACCTTTACCAGTGGAATGACG	TGGGTCTCTTCAGGTTGGTTTG
**H1c pre-mRNA**	GAGCCACCACTCCCACTTAAG	GGATCGAGTCCCTTGCAAC
**hSMN2 Total**	GTGAGGCGTATGTGGCAAAAT	CATATAGAAGATAGAAAAAAACAGTACAATGACC
**Smn mRNA**	TGCTCCGTGGACCTCATTTCTT	TGGCTTTCCTGGTCCTAATCCTGA
**SMN2 FL**	CACCACCTCCCATATGTCCAGATT	GAATGTGAGCACCTTCCTTCTTT
**Stasimon Aberrant**	TGACGCCAAGGCTCTAGGAAAA	CCAAGTCCGGAGCATTGTACATAAAAGG

Supplementary Table 3 List of primer sets used for RT-qPCR
